# The antibacterial mechanism of oridonin against methicillin-resistant *Staphylococcus aureus* (MRSA)

**DOI:** 10.1080/13880209.2019.1674342

**Published:** 2019-10-17

**Authors:** Zhongwei Yuan, Ping Ouyang, Kexin Gu, Tayyab Rehman, Tianyi Zhang, Zhongqiong Yin, Hualin Fu, Juchun Lin, Changliang He, Gang Shu, Xiaoxia Liang, Zhixiang Yuan, Xu Song, Lixia Li, Yuanfeng Zou, Lizi Yin

**Affiliations:** College of Veterinary Medicine, Sichuan Agriculture University, Chengdu, China

**Keywords:** Bacteriostat, cell membrane, cell wall, protein metabolism, LDH, DNA, microscopic structure

## Abstract

**Context:** Methicillin-resistant *Staphylococcus aureus* (MRSA) is a very harmful bacterium. Oridonin, a component in *Rabdosia rubescens* (Hemsl.) Hara (Lamiaceae), is widely used against bacterial infections in China.

**Objective:** We evaluated oridonin effects on MRSA cell membrane and wall, protein metabolism, lactate dehydrogenase (LDH), DNA and microscopic structure.

**Materials and methods:** Broth microdilution and flat colony counting methods were used to measure oridonin minimal inhibitory concentration (MIC) and minimum bactericidal concentration (MBC) against USA300 strain. Electrical conductivity and DNA exosmosis were analysed to study oridonin effects (128 μg/mL) on cell membrane and wall for 0, 1, 2, 4 and 6 h. Sodium dodecyl sulphate polyacrylamide gel electrophoresis was used to detect effects on soluble protein synthesis after 6, 10 and 16 h. LDH activity was examined with an enzyme-linked immunosorbent assay. Effects of oridonin on USA300 DNA were investigated with DAPI staining. Morphological changes in MRSA following oridonin treatment were determined with scanning electron microscopy (SEM) and transmission electron microscopy (TEM).

**Results:** Oridonin MIC and MBC values against USA300 were 64 and 512 μg/mL, respectively. The conductivity and DNA exosmosis level of oridonin-treated USA300 improved by 3.20±0.84% and increased by 58.63 ± 1.78 μg/mL, respectively. LDH and soluble protein levels decreased by 30.85±7.69% and 27.51 ± 1.39%, respectively. A decrease in fluorescence intensity was reported with time. Oridonin affected the morphology of USA300.

**Conclusions:** Oridonin antibacterial mechanism was related to changes in cell membrane and cell wall permeability, disturbance in protein and DNA metabolism, and influence on bacterial morphology. Thus, oridonin may help in treating MRSA infection.

## Introduction

*Staphylococcus aureus* is a common Gram-positive bacterium widely distributed in the air, water, dust and human wastes (Lowy 1998). It is a conditional pathogen that may cause various infectious diseases such as pneumonia, peritonitis and bacteraemia in immunocompromised humans and animals (Foster et al. 2014; Rodvold and McConeghy 2014). The wide, irrational use of antibiotics for bacterial infections has deemed *S. aureus* resistant and led to the emergence of clinically challenging strains, especially methicillin-resistant *Staphylococcus aureus* (MRSA) and vancomycin-resistant *Staphylococcus aureus* (VRSA) (Amalaradjou and Venkitanarayanan 2014). MRSA infection is one of the three most intractable infectious diseases, including hepatitis B and acquired immunodeficiency syndrome, in the world (Cho and Chung 2017). MRSA is one of the most common pathogenic bacterium in nosocomial infections (Lindsay 2013). The percentage of MRSA among *S. aureus* is increasing year-by-year (Bogomolova et al. 2011). MRSA has emerged as a serious threat to public health worldwide, as the infections caused by this bacterium are associated with higher morbidity and mortality and accrue higher therapy costs than normal *S. aureus* infections (Dryden 2008). Center for Disease Control and Prevention (CDC) has reported more than 19,000 deaths in hospitals owing to MRSA infections every year in the United States (Klevens et al. 2007). Thus, there is an urgent need for new drugs specific for MRSA infections. USA300, a representative MRSA strain (van der Mee-Marquet et al. 2015; Bouchiat et al. 2017; Immergluck et al. 2017), was chosen as the strain of interest in this study.

Compounds isolated from herbal medicines have been widely used for the treatment of microbial infections for several decades (Barbieri et al. 2017). As a traditional Chinese medicine, *Rabdosia rubescens* (Hemsl.) Hara (Lamiaceae) plays an important role in heat resistance, detoxification, blood activation and pain alleviation (Yang et al. 2017). In modern medicine, oridonin is known for its potent antimicrobial, antioxidative, anti-inflammatory and pharmacological activities (Weiyan et al. 2017). Oridonin was shown to inhibit the growth of several bacteria, including *Salmonella typhi*, *Streptococcus pneumonia*, *Shigella castellani* and *S. aureus* (Li et al. 2016). In addition, a pilot experiment revealed the antibacterial activity of oridonin against MRSA. However, the mechanism underlying its antibacterial property against MRSA is yet unknown.

In the present study, we evaluated the underlying mechanism of action of oridonin against MRSA by studying its inhibitory effects on cell membrane, cell wall, protein metabolism, lactate dehydrogenase (LDH), DNA and microscopic structure.

## Materials and methods

### Bacterial strain and drug reagent

The MRSA strain USA300 (ATCC BAA-1717) (obtained from the American Type Culture Collection (ATCC)) was used in the present study and cultivated on brain heart infusion (BHI) broth (Sigma, St. Louis, MO). Oridonin (>98% HPLC purity; CAS no. 28957-04-2) was commercially obtained from Chengdu Herbpurify Co., Ltd. (Chengdu, China) and dissolved in dimethyl sulphoxide (DMSO) to obtain a stock solution.

### Susceptibility testing and growth curve assay

The broth microdilution method was used to measure the minimal inhibitory concentration (MIC) of oridonin against USA300 as per the guidelines of the Clinical and Laboratory Standards Institute (Arendrup et al. 2017). The lowest concentration of oridonin that inhibited the growth of USA300 was defined as the MIC. The minimum bactericidal concentration (MBC) value against USA300 was determined with the BHI plate method (Wilson et al. 2018). Control experiment was carried out using a fixed concentration of DMSO in duplicates. To examine growth curves of USA300 following exposure to oridonin, 5 mL overnight bacterial cultures were added to 500 mL fresh BHI broth. After the culture absorbance at 600 nm (OD_600 nm_) reached 0.3, the bacterial cultures were treated with oridonin at final concentrations of 0, 8, 16, 32, 64 and 128 μg/mL. The cultures were incubated under constant shaking (200 rpm) at 37 °C (Ouyang et al. 2017). The OD_600 nm_ value of the culture was measured at regular intervals with a UV-spectrophotometer (UV-2000, UNICO, Shanghai, China), and the growth curve was plotted according to the absorbance value. The experiments were repeated three times.

### Electrical conductivity assay

Effects of oridonin on USA300 membrane permeability were determined by measuring electrical conductivity (Zhang et al. 2018). After 16 h of cultivation, the BHI broth containing USA300 cells was treated with oridonin for 0, 1, 2, 4 and 6 h. The supernatant was collected after centrifugation of cultures at 4500 rpm for 10 min, and diluted 20 times with 5% glucose solution. Electrical conductivity was immediately determined by a conductivity metre (Lee et al. 1998). To increase accuracy, the experiments were repeated three times.

### Measurement of DNA content

USA300 strain was cultivated in BHI broth at 200 rpm and 37 °C. Once the OD_600 nm_ value reached 0.8, the cells were collected by centrifugation (4500 rpm and 10 min) and the pellet was washed twice with sterile phosphate-buffered saline (PBS). The pellet was resuspended in PBS at a concentration of 10^7^ CFU/mL. Oridonin was added to the bacterial suspension at a final concentration of 128 μg/mL, and the cells were incubated at 37 °C without shaking. The supernatant was collected by centrifugation (5000 rpm and 10 min) after 0, 1, 2, 4 and 6 h. The content of DNA in the supernatant liquid was determined with a micro-spectrophotometer (NanoDrop One, Thermo Scientific, Waltham, MA) (Chen and Cooper 2002). The experiments were repeated three times with appropriate untreated controls.

### Protein metabolism assay

The overnight cultivated USA300 cells were inoculated into fresh BHI broth (1:100). The cells were cultured until the OD_600 nm_ value reached 0.8, followed by their treatment with oridonin at a final concentration of 128 μg/mL. The cultures were incubated in a chamber at 37 °C and 200 rpm. After 6, 10 and 16 h of cultivation, the bacterial cells were obtained by centrifugation (4500 rpm and 10 min). The cells were resuspended in sterile PBS and subjected to sonication (VCX750, Sonics, Newtown, CT). The protein content was determined with bicinchoninic acid (BCA) Protein Assay Kit (P0010S, Beyotime Biotechnology, Nanjing, China). In total, 80 μL cell mixture was boiled with 20 μL a 5× loading buffer. After centrifugation at 4500 rpm for 10 min, the mixture was separated with sodium dodecyl sulphate polyacrylamide gel electrophoresis (SDS-PAGE) (Wu et al. 2018). The experiment was repeated three times with appropriate untreated control.

### LDH measurement

Oridonin (128 μg/mL) was added to the medium containing USA300 at the logarithmic phase (OD_600 nm_ = 0.6). After incubation for 1, 2, 4 and 8 h, the samples were centrifuged at 5500 rpm for 10 min. The bacterial cells were collected, resuspended in sterile PBS, and subjected to ultrasonication. The concentration of LDH was measured using an LDH assay kit (Jiancheng Bioengineering, Nanjing, China).

### 4′,6-Diamidino-2-phenylindole (DAPI) staining

Oridonin (128 μg/mL) was added to the bacterial culture (OD_600 nm_ = 0.4). After 0, 2, 4 and 8 h of incubation at 37 °C, the bacterial cells were fixed on a microscope slide and treated with DAPI. The cells were incubated at 25 °C for 20 min in the dark and the DNA fluorescence was observed under a fluorescence microscope (OLYMPUS, Tokyo, Japan).

### Cell morphology analysis with scanning electron microscopy (SEM) and transmission electron microscopy (TEM)

USA300 was cultivated in BHI broth until an OD_600 nm_ value of 0.8 at 200 rpm and 37 °C (Nanda et al. 2016) and then treated with oridonin (128 μg/mL). After 1 h, the bacterial cells were collected by centrifugation (4500 rpm and 10 min) and used for SEM analysis, as previously described (Lin et al. 2015; He et al. 2018; Ma et al. 2019). For TEM analysis, the cells were collected after 16 h incubation and prepared as appropriate (Myklebust et al. 1975). Control experiments were performed without oridonin treatment.

### Statistical analysis

Statistical significance was analysed with GraphPad Prism 7 software (GraphPad Software, La Jolla, CA) using the unpaired two-tailed Student’s *t*-test or deviation analysis. Data are presented as the mean ± standard error of mean (SEM). Differences with *p*< 0.05 were considered statistically significant.

## Results

### Oridonin affected the growth of USA300

**Figure 1.**
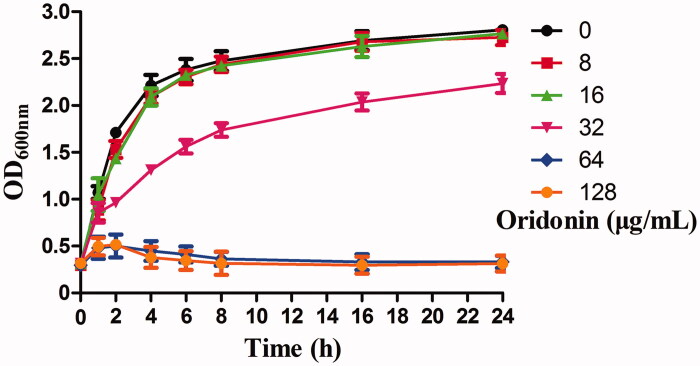
Growth curves of USA300 in the presence of different concentrations of oridonin in BHI.

The MIC of oridonin against MRSA strain (USA300) was 64 μg/mL, while the MBC value was 512 μg/mL. Analysis of growth curves (Figure 1) showed that oridonin had no inhibitory effect on USA300 growth at concentrations of 8 and 16 μg/mL. However, oridonin exerted inhibitory activity against USA300 strain at 32 μg/mL concentration.

### Oridonin increased the membrane permeability of USA300

**Figure 2.**
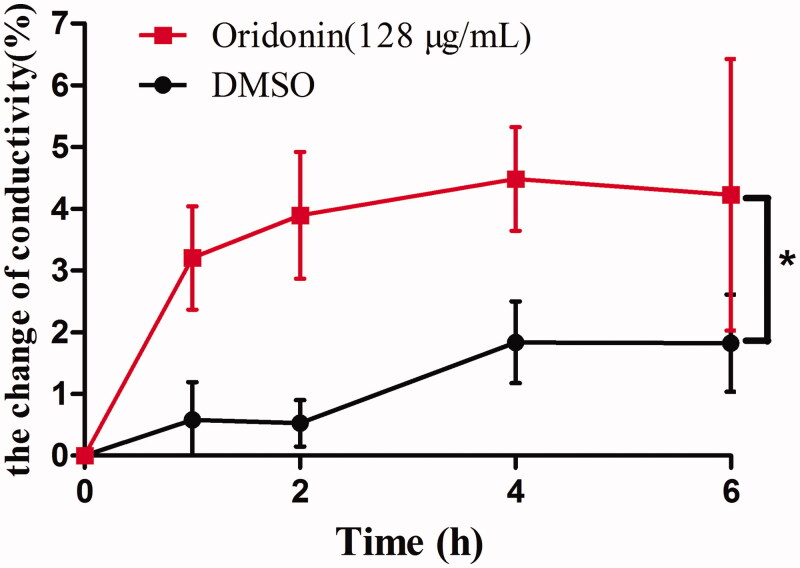
Effect of oridonin (128 μg/mL) on the conductivity of MRSA (oridonin group versus DMSO group, *p < 0.05).

To investigate the effects of oridonin on USA300 cell membrane, cell conductivity was measured. Figure 2 shows that oridonin enhanced the conductivity of the bacterial solution. At 128 μg/mL concentration, oridonin treatment for 1 h improved the conductivity by 3.20±0.84% (*p* < 0.05) as compared to that observed at 0 h. After 2 h of treatment, the conductivity improved by 3.89±1.03% (*p* < 0.05). The change in the conductivity of the control group was only 0.58±0.61% (*p* > 0.05) after 1 h. The change in the conductivity of control group was not obvious especially in the first 2 h.

### Oridonin increased the cell wall permeability of USA300

**Figure 3.**
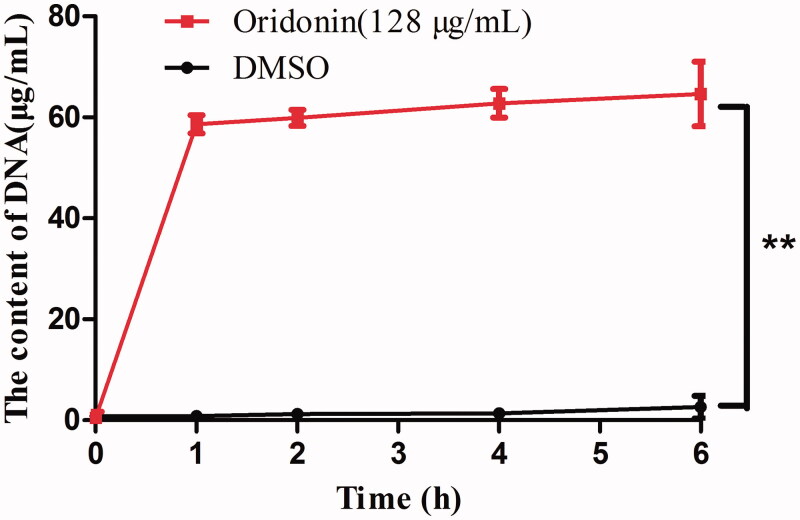
Effects of oridonin (128 μg/mL) on the permeability of USA300 cell wall (oridonin group versus DMSO group, **p < 0.01).

To investigate the effect of oridonin on USA300 cell wall, the extracellular DNA content was measured. Oridonin increased the content of extracellular DNA (Figure 3). After 1 h, the content of DNA increased by 58.63 ± 1.78 μg/mL (*p* < 0.01) for oridonin-treated group as compared with that observed for the control group. Thus, oridonin could change the permeability of USA300 cell wall.

### Oridonin affected the protein metabolism of USA300

**Figure 4.**
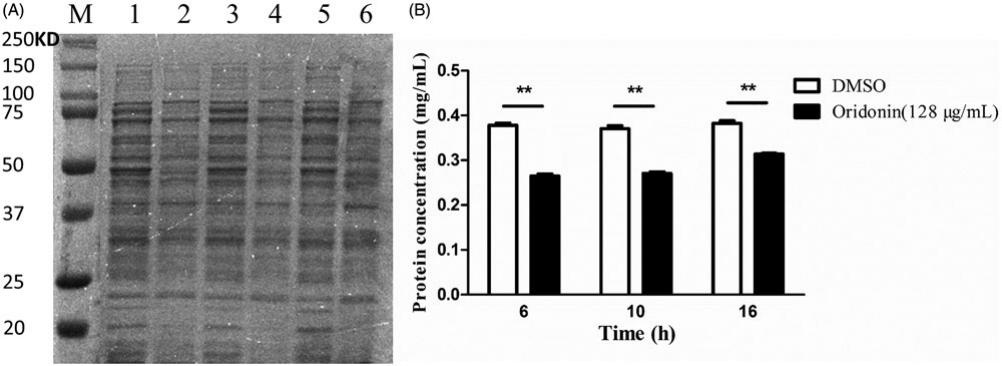
Effect of oridonin (128 μg/mL) on the protein metabolism of USA300. (A) SDS-PAGE image. (B) Results of BCA protein assay kit. Lane M, maker; lanes 1, 3 and 5, untreated cells for 6, 10 and 16 h, respectively; lanes 2, 4 and 6, treated cells with 128 μg/mL for 6, 10 and 16 h, respectively. **p < 0.01 as compared with DMSO group.

The results of SDS-PAGE revealed the effects of oridonin on the metabolism of several soluble proteins from USA300. The protein profile changed after the treatment of the bacterium with oridonin for 6, 12 and 16 h. Clear and bright protein bands (Figure 4(A), lanes 1, 3 and 5) were detected in the control group. However, the treatment group (Figure 4(A), lanes 2, 4 and 6) showed lighter bands than the control group (without oridonin). After 10 h, the total amount of soluble protein in the drug treatment group decreased by 27.51±1.39% (*p* < 0.01) as compared with that in the control group (Figure 4(B)). Figure 4 indicates that oridonin inhibited the growth of MRSA by affecting the metabolism of soluble protein.

### Oridonin decreased the LDH activity of USA300

**Figure 5.**
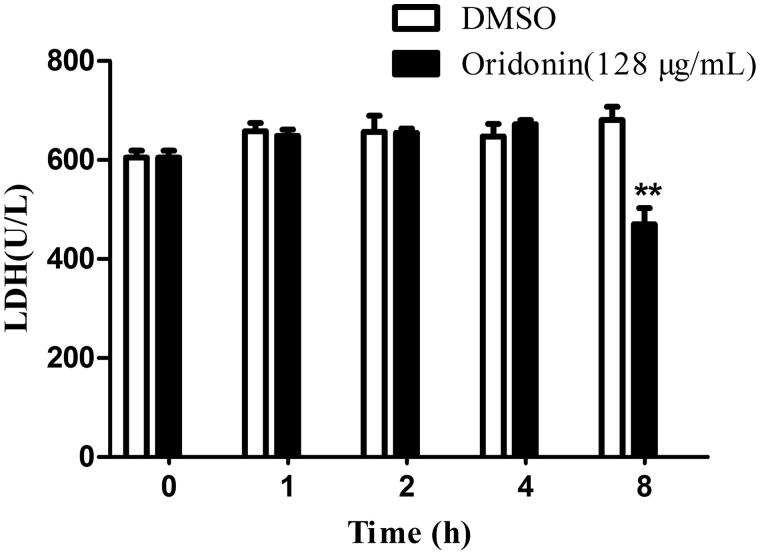
Effect of oridonin (128 μg/mL) on the LDH level of USA300. **p < 0.01 as compared with DMSO group.

Results of LDH analysis are shown in Figure 5. The LDH content showed no significant change after 4 h of treatment with oridonin. After 8 h, the LDH content of the group treated with oridonin decreased by 30.85±7.69% (*p* < 0.01). Thus, the LDH activity of USA300 was inhibited by oridonin in a time-dependent manner.

### Effect of oridonin on DNA

**Figure 6.**
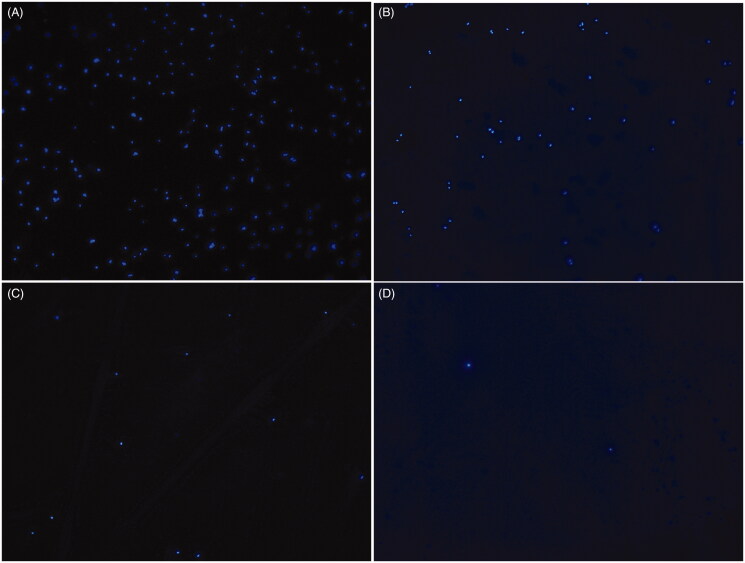
Fluorescence intensity of USA300 DNA. (A) Untreated cells. (B) Cells treated with oridonin (128 μg/mL) for 2 h. (C) Cells treated with oridonin (128 μg/mL) for 4 h. (D) Cells treated with oridonin (128 μg/mL) for 8 h.

Figure 6(A) shows high fluorescence intensity for the control group. The fluorescence intensity decreased as the density was lower with an increase in the drug treatment time (Figure 6(B–D)).

### Effect of oridonin on cell morphology

**Figure 7.**
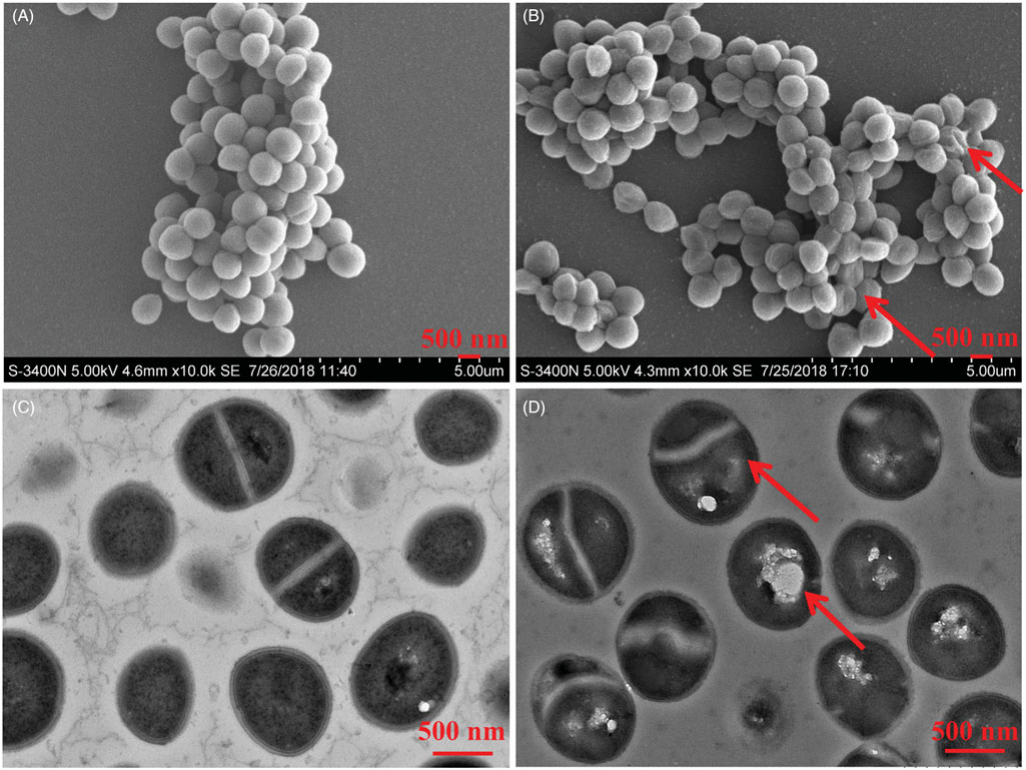
Morphology of USA300 cells (×10,000) as examined under a scanning electron microscope (SEM) and transmission electron microscope (TEM). (A) Untreated USA300 cells after 1 h (SEM). (B) USA300 cells treated with oridonin (128 μg/mL) for 1 h (SEM). (C) Untreated USA300 cells after 16 h (TEM). (D) USA300 cells treated with oridonin (128 μg/mL) for 16 h (TEM).

SEM and TEM images of USA300 treated with or without oridonin are shown in Figure 7. The untreated cells showed smooth, round and spherical surfaces with clear boundaries between them; the cells were arranged in clusters of grapes under SEM (Figure 7(A)). However, the arrangement of cells was loose and irregular, and the bacterial cells failed to form clusters in the treated group. The boundaries between the cells were slightly blurred, and some bacteria formed clumps. The blurring of cell boundaries was indicative of the damaged cell walls. The biodegradable cell wall polysaccharides gathered around the bacterial cells, thereby inducing clump formation. Some cells were deformed (Figure 7(B)). The results of TEM are shown in Figure 7(C,D). The cell structure was complete and clear and the cell wall was smooth in the control group (Figure 7(C)). The cytoplasm and nuclear area were evident and the septa of cell division were normally visible. The cells from the treatment group, on the other hand, showed loss of intracellular material, cytoplasmic condensation, obvious vacuolation, blurry constriction and abnormal binary fission.

## Discussion

The antibacterial mechanism of action of several chemical compounds isolated from plants is associated with cell membrane permeability (Dorman and Deans 2000). In bacteria, cell membrane is a selective permeability barrier. Bacterial cell membrane can protect the bacterium from harmful compounds such as drugs, toxins, detergents and degradative enzymes and allows penetration of nutrients for bacterial growth (Strahl and Errington 2017). Cell membrane integrity affects the life activities of bacteria. In the present study, the conductivity assay result revealed the rapid increase in the conductivity of bacterial solution in 1 h from oridonin treatment. Thus, oridonin could increase the permeability of bacterial cell membrane, thereby inducing cytoplasmic leakage in a short time.

Several microscopic pores in the bacterial cell wall allow movement of water and small molecules. However, bacterial cell wall is only slightly permeable to biomolecules such as DNA. Cell membranes also restrict the movement of large molecules of DNA. The results of conductivity test showed that oridonin increased the DNA content in the solution, suggestive of the oridonin-mediated destruction of the structure of bacterial cell membrane.

Proteins play important roles in living organisms by supplying energy, catalysing metabolic reactions and participating in immune responses (Typas and Sourjik 2015). Soluble proteins are important osmotic regulators in bacteria (Meyer and Cusanovich 1989; Fairhead et al. 1994). The decrease in the content of soluble proteins could reduce the water retention ability of cells. The results of SDS-PAGE showed a significant decrease in the protein content of the bacteria treated with oridonin. Oridonin affected bacterial growth and propagation by interfering with the metabolism of proteins.

LDH is an important enzyme that catalyses the mutual transformation of pyruvate and lactic acid. It is essential for MRSA to maintain virulence, evade the innate immunity of the host, and form biofilm (Ferreira et al. 2013). LDH assay showed that oridonin could reduce the synthesis of LDH. Thus, oridonin has the potential to influence MRSA toxicity and improve the host immune response. LDH is an essential soluble protein, and the change in its level was consistent with the results of soluble protein assay.

DNA, a relatively stable genetic material, directs protein synthesis and controls metabolism. DAPI staining assay is an effective method to investigate the effect of oridonin on USA300 DNA. According to a previous study, abnormal protein metabolism may be associated with the decrease in the DNA content (Han et al. 2007). In the present study, the decrease in the DNA content after 2 h of drug treatment may be attributed to the rupture of cell membranes. A small amount of outflow DNA in the cytoplasm led to a weak fluorescence intensity and low fluorescence density. After 2 h, the fluorescence intensity reduced owing to the oridonin-mediated damage to the DNA in the nucleoid.

SEM and TEM are widely used for the study of bacteriostatic mechanism. The results of SEM showed that cell wall and membrane of USA300 strain were disrupted. Oridonin exerted antibacterial activities on MRSA, probably through the loss of the cytoplasmic material in response to the damage to the cell structure. The results of TEM revealed the effect on the fission of USA300 cells. Oridonin may affect protein metabolism and consequently impact bacterial fission.

## Conclusions

The results of the present study demonstrate the significant antibacterial activity of oridonin at 128 μg/mL concentration against MRSA. This compound affected the permeability of bacterial cell membrane and cell wall, protein metabolism, as well as cellular metabolism and morphology. Thus, oridonin exhibits prominent antibacterial activity against MRSA *in vitro*. It is imperative to perform future studies involving structural modifications and dose response to meet clinical needs.
